# Analysis of Bovine Viral Diarrhea Viruses-infected monocytes: identification of cytopathic and non-cytopathic biotype differences

**DOI:** 10.1186/1471-2105-11-S6-S9

**Published:** 2010-10-07

**Authors:** Mais Ammari, Fiona M McCarthy, Bindu Nanduri, Lesya M Pinchuk

**Affiliations:** 1 Department of Basic Sciences, Mississippi State University, Mississippi State, MS 39762, USA; 2 Institute of Digital Biology, Mississippi State University, Mississippi State, MS 39762, USA

## Abstract

**Background:**

Bovine Viral Diarrhea Virus (BVDV) infection is widespread in cattle worldwide, causing important economic losses. Pathogenesis of the disease caused by BVDV is complex, as each BVDV strain has two biotypes: non-cytopathic (ncp) and cytopathic (cp). BVDV can cause a persistent latent infection and immune suppression if animals are infected with an ncp biotype during early gestation, followed by a subsequent infection of the cp biotype. The molecular mechanisms that underscore the complex disease etiology leading to immune suppression in cattle caused by BVDV are not well understood.

**Results:**

Using proteomics, we evaluated the effect of cp and ncp BVDV infection of bovine monocytes to determine their role in viral immune suppression and uncontrolled inflammation. Proteins were isolated by differential detergent fractionation and identified by 2D-LC ESI MS/MS. We identified 137 and 228 significantly altered bovine proteins due to ncp and cp BVDV infection, respectively. Functional analysis of these proteins using the Gene Ontology (GO) showed multiple under- and over- represented GO functions in molecular function, biological process and cellular component between the two BVDV biotypes. Analysis of the top immunological pathways affected by BVDV infection revealed that pathways representing macropinocytosis signalling, virus entry via endocytic pathway, integrin signalling and primary immunodeficiency signalling were identified only in ncp BVDV-infected monocytes. In contrast, pathways like actin cytoskeleton signalling, RhoA signalling, clathrin-mediated endocytosis signalling and interferon signalling were identified only in cp BDVD-infected cells. Of the six common pathways involved in cp and ncp BVDV infection, acute phase response signalling was the most significant for both BVDV biotypes. Although, most shared altered host proteins between both BVDV biotypes showed the same type of change, integrin alpha 2b (ITGA2B) and integrin beta 3 (ITGB3) were down- regulated by ncp BVDV and up- regulated by cp BVDV infection.

**Conclusions:**

This study shows that, as we expected, there are significant functional differences in the host proteins that respond to cp or ncp BVDV infection. The combined use of GO and systems biology network modelling facilitated a better understanding of host-pathogen interactions.

## Background

Bovine Viral Diarrhea Virus (BVDV) infections are seen in all ages and breeds of cattle worldwide and have significant economic impact due to productive and reproductive losses [1,2]. BVDV is a single-stranded, positive-sense RNA virus, belonging to the* Flaviviridae* family, genus* Pestivirus *[1,2]. BVDV genotypes are classified according to their effects in cell cultures into two different biotypes: non-cytopathic (ncp) and cytopathic (cp). Different isolates of both forms commonly exhibit antigenic differences [3,4]. The pathogenesis of the disease caused by BVDV is complex and involves persistent infection (PI) and immune suppression with the ncp biotype during early gestation, followed by an acute infection by a cp biotype [5,6]. PI animals shed virus and initiate further virus replication and genetic variation [5,6]. The fatal form of BVDV mucosal disease only occurs in animals carrying the ncp biotype and become exposed to the cp biotype [6]. Even though BVDV is one of the most studied infective agents in cattle it is probably one of the least understood. This is mainly because BVDV are a group of multiple viruses affecting virtually all organs and system in the body, including innate and adaptive immune system [7]. Identifying the molecular mechanisms and developing strategies for controlling the spread of the virus are the challenges faced by BVDV researchers.

Taking into consideration that PI animals are the major disseminators of BVDV in the cattle population, we hypothesized that low doses of BVDV infection can provide some answers in the BVDV pathogenesis. In our earlier work we assessed selective and non-selective antigen uptake mechanisms in BVDV-infected monocytes and outlined some similarities and differences between the two BVDV biotypes [8]. Following the differences in the antigen uptake function of monocytes and using the same infection protocols we determined the TLR, cytokine and costimulatory molecules gene expression in the infected cells [9]. Francini et al. using high doses of BVDV* in vitro* did not detect significant differences in the TLR expression levels in bovine macrophages [10]. Using a proteomic approach, we demonstrated that cp BVDV biotype affected the expression of proteins related to professional antigen presentation. In particular, proteins related to immune responses, such as cell adhesion, apoptosis, antigen uptake, processing and presentation, acute phase response proteins, MHC class I- and class II-related proteins and other molecules involved in immune function of professional antigen presentation have been significantly altered after BVDV infection [9]. Finally, we demonstrated the differential effects of cp and ncp BVDV biotypes on the expression levels of the protein kinases and related proteins affecting the development of infection and antiviral mechanisms in bovine monocytes [11].

To better understand the complexity of the mechanisms by which the cp and ncp BVDV cause disease, and to identify biotype-related differences in significant biological functions and pathways here we further analyzed the expression of immunologically important proteins by combined use of GO and systems biology network modelling.

## Results

**Figure 1:**
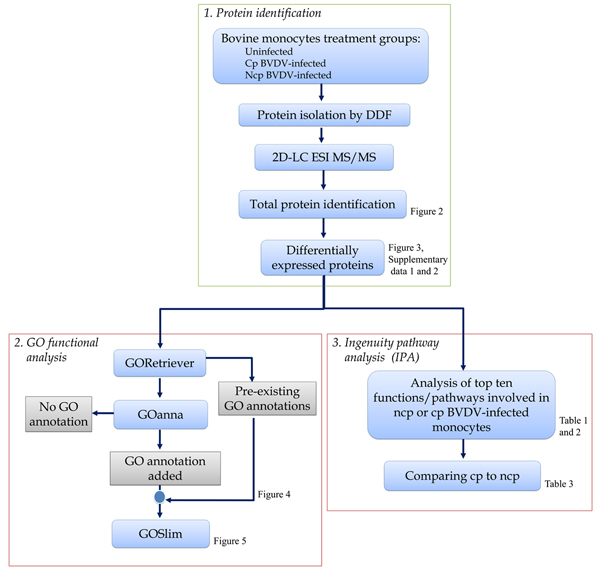
**Identification and analysis of protein profiles of bovine monocytes infected with cp and ncp BVDV biotypes.** Process flowchart showing the major steps in bovine monocytes infection, protein isolation, identification and analysis with corresponding figures and tables.

Our overall approach to determine the differential effects of cp and ncp BVDV infection on the monocyte-dependent innate and adaptive immune responses involved identification of differentially expressed proteins in each type of infection followed by functional modelling using both GO and Ingenuity Pathway Analysis (IPA) pathway and network analysis (Figure 1). The results of each of these steps are presented in more detail in the following sections.

### 1. Protein identification and differentially expressed proteins in ncp and cp BVDV-infected monocytes

**Figure 2:**
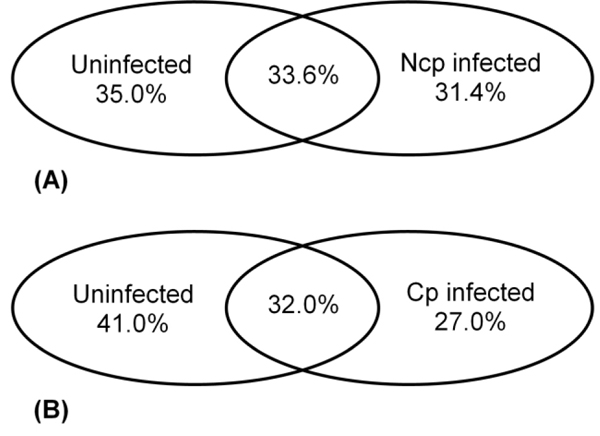
**Distribution of total proteins identified in BVDV-infected monocytes compared to uninfected controls** Using 2D-LC ESI MS/MS approach a total of 2489, 2356 and 2028 bovine proteins were identified in uninfected, ncp and cp BVDV infected bovine monocytes within two replicates respectively. Distribution of identified proteins compared to uninfected monocytes is shown for (A) ncp infection; and (B) cp infection.

We initially identified a total of 2489, 2356 and 2028 bovine proteins from uninfected, ncp and cp BVDV-infected bovine monocytes, respectively. By comparing ncp BVDV-infected host proteins to their uninfected counterparts we were able to determine up- and down- regulated host proteins occurring in either cp or ncp BVDV infection (Figure 2). This gave us a total of 1137 (31.4%) altered proteins unique to ncp BVDV-infected monocytes and 929 (27.0%) altered proteins unique to cp BVDV-infected cells.

**Figure 3:**
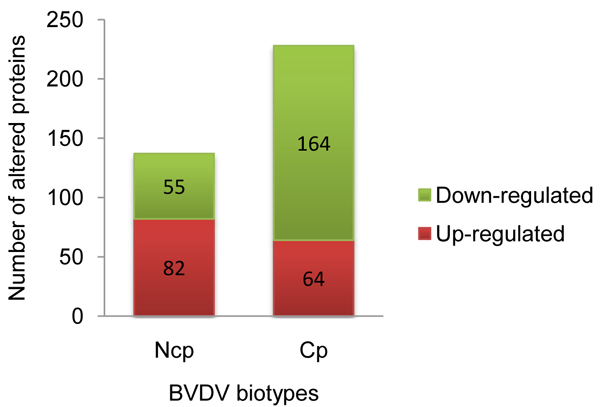
**Differentially expressed host proteins in BVDV-infected monocytes compared to uninfected controls** Compared to uninfected monocytes, ncp infection differentially alters the expression of 137 host proteins with 40.2% down-regulated proteins and 59.8% up-regulated proteins. Whereas, compared to uninfected monocytes, cp infection alters the expression of 228 host proteins with increasing the percentage of down-regulated proteins to 72% and decreasing the percentage of up-regulated proteins to 28% compared to the effect of ncp on uninfected monocytes.

Compared to uninfected monocytes, ncp BVDV altered the expression of 137 host proteins with 55 (40.2%) being down-regulated and 82 (59.8%) being up-regulated (Figure 3, additional file 1). In comparison, cp BVDV altered the expression of 228 host proteins of which 164 (72.0%) were down-regulated and 64 (28.0%) were up-regulated, compared to uninfected monocytes (Figure 3, additional file 2). Of these differentially expressed proteins, 69 host proteins were common to ncp and cp BVDV infections. The expression trends for these shared proteins were similar for all except for integrin alpha 2b (ITGA2B) and integrin beta 3 (ITGB3), that were down- regulated by ncp BVDV and up- regulated by cp BVDV infection.

Comparison of proteins unique to ncp BVDV-infected monocytes (1137) with proteins unique to cp BVDV-infected cells (929) showed that 240 (13.2%) common host response proteins, 897 (49.1%) and 689 (37.7%) proteins unique to ncp and cp BDVD-infected monocytes, respectively (data not shown).

### 2. GO Functional analysis of BVDV-infected monocytes

**Figure 4:**
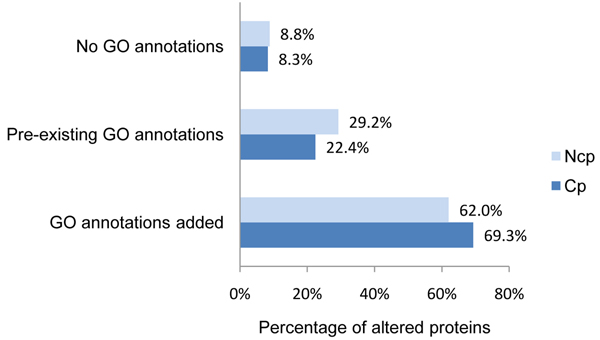
**GO annotation for differentially expressed proteins in BVDV-infected monocytes** The numbers of GO annotated bovine proteins are shown as a percentage. Since only 22-29% initially had GO annotation available, we added our own additional GO annotations. In total we obtained GO annotation for 91.2% and 91.7% of our differentially altered host response proteins due to ncp and cp BVDV-infection, respectively.

**Figure 5:**
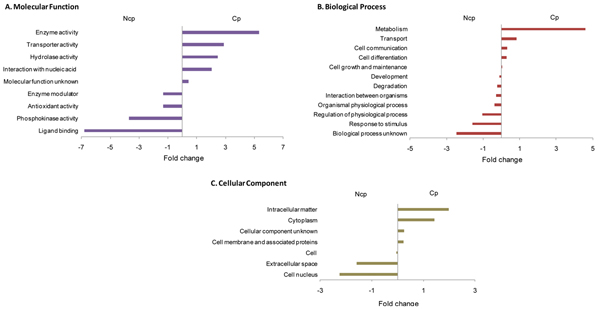
**GO functional analysis of host proteins differentially expressed during ncp or cp BVDV-infected monocytes** The cp and ncp host proteins that were differentially expressed compared to uninfected monocytes were functionally analyzed by summarizing GO functional annotations based on the GOA and Whole Proteome GOSlim set and the results were compared to obtain the relative differences between the two biotypes. The top under- and over- represented GO functions are shown here: A. Molecular Functions (MF), B. Biological Process (BP) and C. Cellular Components (CC). Subcategories within functional groups are listed on the y-axis and the relative percentage difference in the numbers of proteins altered by cp and ncp is the x-axis.

GO annotations were publicly available for 29.2% and 22.4% of the bovine proteins in our ncp and cp BVDV datasets, respectively. We further assigned GO annotations to an additional 62.0% and 69.3% of bovine proteins, respectively; bringing the total number of proteins with GO annotation available for functional analysis to 91.2% and 91.7 % of our ncp and cp BVDV datasets, respectively (Figure 4). This enabled us to perform a comprehensive GO functional modelling. Our GO annotations have been submitted to AgBase, where they will be quality checked and made publicly available. We summarized the GO annotations for bovine proteins differentially expressed in cp and ncp BVDV infections to identify biological functions in the host response that correspond to infections with these two biotypes. Antioxidant activity, ligand binding, response to stimulus, and extracellular space were over-represented in the ncp BVDV-infected monocytes compared to their cp BVDV-infected counterparts (Figure 5). Transport, enzyme activity, metabolism, and intracellular matters are more highly represented during cp BVDV infection (Figure 5).

### 3. Proteins with significantly altered expression in cp and ncp BVDV-infected monocytes: network and pathway analysis

At IPA threshold of significance, 6 and 4 networks and 42 and 33 functions/diseases were significantly represented in the proteomes of ncp and cp BVDV-infected monocytes, respectively. The top ten functions/diseases (ranked based on significance), and the associated signalling pathways are shown in Tables 1 and 2. Analysis of the top ten pathways revealed that pathways representing macropinocytosis signalling, virus entry via endocytic pathway, integrin signalling and primary immunodeficiency signalling were identified only in ncp BVDV-infected monocytes. In contrast, pathways like actin cytoskeleton signalling, RhoA signalling, clathrin-mediated endocytosis signalling and interferon signalling were identified only in cp BVDV-infected cells. Of the six common pathways involved in cp and ncp BVDV infection, acute phase response signalling was the most significant for both BVDV biotypes (Tables 1, 2). In each of those six pathways, cp BVDV altered the expression of 33 host proteins compared to the 24 altered proteins due to ncp BVDV infection.

**Table 1:** Top ten functions/diseases and their respective top ten pathways in ncp BVDV- infected monocytes

	Acute phase response signaling (8)	Caveolae- mediated endocytosis signaling (5)	B cell development (3)	IL-10 signaling(3)	Macropinocytosis signaling (3)	Virus entry via endocytic pathway (3)	Antigen presentation pathway (2)	Fcy receptor mediated phagocytosis in macrophages and monocytes (3)	Integrin signaling (4)	Primary immunodeficiency signaling (2)
**Cellular movement (10)**		ITGB3	SPN	–	CSF1R, ITGB3	ITGB3	–	FGR	ITGB3	–
**Immune cell trafficking (18)**	–	ITGA6, ITGB3	SPN, PTPRC	CD14	CSF1R, ITGB3, CD14	ITGA6, ITGB3	–	FGR	ITGA6, ITGB3	PTPRC
**Inflammatory response(20)**	TF	ITGA6, ITGB3	SPN, PTPRC	CD14	CSF1R, ITGB3, CD14	ITGA6, ITGB3	–	FGR	ITGA6, ITGB3	PTPRC
**Cell death (11)**	SOD2	–	PTPRC, HLA- DRB1	CD14	CSF1R, ITGB3	–	HLA-DRB1	FGR		PTPRC
**Hematological systemdevelopment and function (21)**	TF	ITGA6, ITGB3	SPN, PTPRC, HLA-DRB1	CD14	CSF1R, ITGB3, CD14	ITGA6, ITGB3	–	FGR	ITGA6, ITGB3	PTPRC
**Cell-to-cell signaling and interaction (10)**	–	ITGB3	PTPRC	CD14	CSF1R, ITGB3, CD14	ITGB3	–	FGR	ITGB3	PTPRC
**Cellular function and maintenance (5)**	–	ITGB3	–	–	ITGB3	ITGB3	–	FGR	ITGB3	–
**Immunologicaldisease (5)**	–	–	PTPRC	CD14	CD14	–	–	–	–	PTPRC
**Antigenpresentation (13)**	A2M	–	PTPRC	CD14	CSF1R, CD14	–	–	FGR	–	–
**Inflammatorydisease (5)**	–	ITGB3	PTPRC	–	ITGB3	ITGB3	–	–	ITGB3	PTPRC

TF: Transferrin; SOD2: Superoxide Dismutase 2, mitochondrial; A2M: Alpha-2-Macroglobulin; ITGB6: Integrin, Beta 6; ITGB3: Integrin, Beta 3 (platelet glycoprotein IIIa, antigen CD61); SPN: Sialophorin; PTPRC: Protein Tyrosine Phosphatase, Receptor type, C; CSF1R: Colony Stimulating Factor 1 Receptor; FGR: Fibrinogen Gamma Chain Numbers between brackets indicates the number of altered proteins involved in the functions/diseases or pathway

**Table 2:** Top ten functions/diseases and their respective top ten pathways in cp BVDV- infected monocytes

	Acute phase response signaling(12)	Fcy receptor mediated phagocytosis in macrophages and monocytes (8)	Actin cytoskeleton signaling (8)	Antigen presentation pathway (3)	B cell development (3)	RhoA signaling(5)	Caveolae- mediated endocytosis signaling (4)	Clathrin- mediated endocytosis signaling (6)	IL-10 signaling(3)	Interferon signaling(2)
**Cellular function and maintenance (7)**	–	FGR, LYN	–	–	–	–	ITGB3	ITGB3	–	–
**cell-to-cell signaling and interaction (8)**	F2	FGR, LYN	CD14, F2	–	–	–	ITGB3	F2, ITGB3	CD14	–
**Inflammatory response (14)**	F2	FGR, LYN	CD14, F2	TAPBP	HLA-DQB1	–	ITGB3	F2, ITGB3	CD14	–
**Cellular assembly and organization (2)**	–	FGR, LYN	–	–	–	–	–	–	–	–
**Protein synthesis (2)**	–	FGR, LYN	–	–	–	–	–	–	–	–
**Cellular compromise (6)**	–	FGR, LYN	–	–	–	–	ITGB3	ITGB3	–	–
**Cell morphology (3)**	–	FGR, LYN	–	–	–	–	–	–	–	–
**Cellular development (4)**	–	FGR, LYN	–	–	–	–	ITGB3	ITGB3	–	–
**Hematological system development and function (9)**	F2	FGR, LYN	CD14, F2	–	SPN	–	ITGB3	F2, ITGB3	CD14	–
**Immune cell trafficking (9)**	F2	FGR, LYN	F2	–	SPN	–	ITGB3	F2, ITGB3	–	–
										

F2: Coagulation Factor II (thrombin); FGR: Fibrinogen Gamma Chain; LYN: v-yes-1 Yamaguchi sarcoma viral related oncogene homolog; TAPBP: TAP Binding protein (Tapasin); SPN: Sialophorin; ITGB3: Integrin, Beta 3 (platelet glycoprotein IIIa, antigen CD61) Numbers between brackets indicates the number of altered proteins involved in the functions/diseases or pathway

Analysis of the ten most significant IPA functions/diseases for the cp and ncp biotypes revealed that five were shared, although different proteins were involved in these pathways. The cp BVDV-altered proteins were involved in five cellular-related functions (Tables 1, 2). When compared, host proteins differentially expressed in cp and ncp BVDV-infected monocytes included acute phase response signalling, Fcγ receptor-mediated phagocytosisin macrophages and monocytes, actin cytoskeleton signalling, antigen presentation pathway, B cell development, RhoA signalling, caveolae-mediated endocytosis signalling, clathrin-mediated endocytosis signalling, IL-10 signalling and interferon signalling (Table 3).

**Table 3:** Differentially altered proteins represented in top ten immunological pathways when comparing cp to ncp BVDV infection

		Regulation		
			
Symbol	Entrez Gene Name	Cp	Ncp	Pathways
**HMOX1**	heme oxygenase (decycling) 1	Up	Up	Acute Phase Response Signaling, Fcy Receptor-mediated Phagocytosis in
				Macrophages and Monocytes, IL-10 Signaling
**ALB**	albumin	Down	Down	Acute Phase Response Signaling, Caveolar-mediated Endocytosis Signaling
**HP**	haptoglobin	Down	Down	Acute Phase Response Signaling
**SOD2**	superoxide dismutase 2, mitochondrial	Up	Up	Acute Phase Response Signaling
**TF**	transferrin	Up	Up	Acute Phase Response Signaling, Clathrin-mediated Endocytosis Signaling
**APOH**	apolipoprotein H (beta-2-glycoprotein I)	Up	–	Acute Phase Response Signaling
**APOA1**	apolipoprotein A-I	Up	–	Acute Phase Response Signaling
**AHSG**	alpha-2-HS-glycoprotein	Up	Up	Acute Phase Response Signaling
**SERPINA1**	serpin peptidase inhibitor, clade A (alpha-1	Up	Up	Acute Phase Response Signaling
	antiproteinase, antitrypsin), member 1			
**A2M**	alpha-2-macroglobulin	Up	Up	Acute Phase Response Signaling
**FGG**	fibrinogen gamma chain	Up	–	Acute Phase Response Signaling
**F2**	coagulation factor II (thrombin)	Up		Acute Phase Response Signaling, Actin Cytoskeleton Signaling, Clathrin-
				mediated Endocytosis Signaling
**ACTR3**	ARP3 actin-related protein 3 homolog (yeast)	Up	Up	Fcy Receptor-mediated Phagocytosis in Macrophages and Monocytes, Actin
				Cytoskeleton Signaling, RhoA Signaling, Clathrin-mediated Endocytosis
				Signaling
**ARPC2**	actin related protein 2/3 complex, subunit 2,	Up		Fcy Receptor-mediated Phagocytosis in Macrophages and Monocytes, Actin
	34kDa			Cytoskeleton Signaling, RhoA Signaling, Clathrin-mediated Endocytosis
				Signaling
**EZR**	ezrin	Down		Fcy Receptor-mediated Phagocytosis in Macrophages and Monocytes, Actin
				Cytoskeleton Signaling, RhoA Signaling
**LYN**	v-yes-1 Yamaguchi sarcoma viral related	Up		Fcy Receptor-mediated Phagocytosis in Macrophages and Monocytes
	oncogene homolog			
**TLN1**	talin 1	Down	–	Fcy Receptor-mediated Phagocytosis in Macrophages and Monocytes
**FYB**	FYN binding protein (FYB-120/130)	Down	–	Fcy Receptor-mediated Phagocytosis in Macrophages and Monocytes
**FGR**	Gardner-Rasheed feline sarcoma viral (v-fgr)	Down	Down	Fcy Receptor-mediated Phagocytosis in Macrophages and Monocytes
	oncogene homolog			
**MYH10**	myosin, heavy chain 10, non-muscle	Up	–	Actin Cytoskeleton Signaling
**MYL6**	myosin, light chain 6, alkali, smooth muscle and	Up		Actin Cytoskeleton Signaling, RhoA Signaling
	non-muscle			
**DIAPH3**	diaphanous homolog 3 (Drosophila)	Down	–	Actin Cytoskeleton Signaling
**CD14**	CD14 molecule	Up	Up	Actin Cytoskeleton Signaling, IL-10 Signaling
**IQGAP1**	IQ motif containing GTPase activating protein 1	_	Up	Actin Cytoskeleton Signaling
**HLA-DRB1**	major histocompatibility complex, class II, DR	Up	Up	Antigen Presentation Pathway, B Cell Development
	beta 1			
**TAP1**	transporter 1, ATP-binding cassette, sub-family	Down	Down	Antigen Presentation Pathway, Interferon Signaling
	B (MDR/TAP)			
**TAPBP**	TAP binding protein (tapasin)	Down	–	Antigen Presentation Pathway
**HLA-DQB1**	major histocompatibility complex, class II, DQ	Down		B Cell Development
	beta 1			
**SPN**	sialophorin	Down	Down	B Cell Development
**PTPRC**	protein tyrosine phosphatase, receptor type, C	_	Up	B Cell Development
**KTN1**	kinectin 1 (kinesin receptor)	Down	Down	RhoA Signaling
**ITGA2B**	integrin, alpha 2b (platelet glycoprotein IIb of	Up	Down	Caveolae-mediated Endocytosis Signaling
	IIb/IIIa complex, antigen CD41)			
**FLNA**	filamin A, alpha	Up	Up	Caveolae-mediated Endocytosis Signaling
**ITGB3**	integrin, beta 3 (platelet glycoprotein IIIa,	Up	Down	Caveolae-mediated Endocytosis Signaling, Clathrin-mediated Endocytosis
	antigen CD61)			Signaling
**ITGA6**	integrin, alpha 6	–	Down	Caveolae-mediated Endocytosis Signaling
**SH3GLB1**	SH3-domain GRB2-like endophilin B1	Down	–	Clathrin-mediated Endocytosis Signaling
**ARG2**	arginase, type II	Up	Up	IL-10 Signaling
**MX1**	myxovirus (influenza virus) resistance 1,	Up		Interferon Signaling
	interferon-inducible protein p78 (mouse)			

## Discussion

The complex and unique nature of BVDV continues to challenge infectious disease researchers, veterinarians, and the cattle industry. In addition to evading the adaptive immune system, BVDV evade key mechanisms of innate immunity [7]. Although a good understanding of the roles of the two biotypes in the production of persistent infections and the precipitation of mucosal disease has been obtained, there are still unanswered questions regarding the origin of cytopathic viruses and the mechanism by which they cause pathological changes in cells.

In our previous studies we used proteomics to identify host proteins involved in professional antigen presentation altered by cp [9] and protein kinases altered by cp and ncp [11] BVDV. We have now extended this work by identifications of altered host proteins by ncp and cp BVDV infection based on rigorous statistical methods for peptide identification and control of false positive identifications. Likewise, the workflow for differential protein expression includes multiple testing corrections [12]. Comparing host proteins in cp and ncp BVDV-infected monocytes to uninfected controls for differential protein expression showed a higher number of affected proteins by cp biotype. In general, cp BVDV showed more profound effect on the protein expression levels in bovine monocytes with significantly increased number of down-regulated proteins and decreased number of up-regulated proteins compared to the ncp BVDV biotype. This observation is in accord with our previous reports that cp BVDV in general, had more profound effects on antigen uptake mechanisms, TLR, cytokine and co-stimulatory molecule gene and protein kinase protein expression levels in bovine monocytes [8,9,11]. The observed significant biotype-related differences might explain the mechanisms by which cp BVDV, in contrast to ncp biotypes that do not induce cell death, cause pathological changes in infected cells, in particular antigen presenting cells.

In contrast to our previous report on the multiple similarities and some significant biotype-related differences in the monocyte protein expression patterns [11], this new complex modelling approach revealed mostly profound biotype-related differences in all functional groups. This observation strongly supports our hypothesis that low doses of BVDV infection can be crucial to understand the complex pathogenesis of BVDV [8,9,11].

Pathway and network analysis of bovine proteins differentially altered by BVDV also identified significant biotype-related differences. It is known that ncp BVD viruses can establish PI as a result of infection of the embryo early in its development by interfering with a key mechanism of the innate immune system through the interferon (INF) type I production [13]. Since INF is also important in the activation of the adaptive immune response, suppression of this signal may be essential for the establishment of PI [13]. We previously reported that both proteins, CD14 and Mx are increased in BVDV-infected monocytes. However, in this study that uses stringent protein identification parameters compared to our earlier proteomics methods, expression of Mx significantly increased with cp BVDV infection only. Mx protein is believed to be induced exclusively via signalling through the type I INF receptor [14,15].

The early stages of the host response to infectious agents include a number of physiological changes, collectively known as the acute phase response. Our previous report identified multiple acute phase response proteins altered by cp BVDV [9]. In this study, acute phase pathway was demonstrated to be the first significant pathway in both ncp and cp BVDV infection. Although, ncp and cp viruses altered different numbers of host proteins in general, they had the same effects on the monocyte protein expression levels. The acute phase response is comprised of reactions localized at the site of infection, as well as the initiation of systemic responses, which include a rapid increase in the serum concentration of some proteins, known as acute phase proteins (APP) [16]. Recently, it is becoming clear that viruses interact with iron metabolism. Iron is needed for virus replication, and therefore, by ensuring the infected cell is iron replete, a virus favours its own growth. Moreover, increased concentrations of iron in the body can cause tissue damage and inflammation and affect organ function [17]. For Hepatitis C viral infection, the detrimental effects of excess iron are well documented, and elevated iron status is also associated with increased mortality in HIV-1 infection [17]. Here we show that both cp and ncp BVDV up-regulate transferrin (TF), a negative acute phase protein and a major iron transporter, causing iron overload and exacerbates disease (an animal with an increased serum transferrin level often suffers from iron deficiency anemia). Alternatively, both ncp and cp BVDV down-regulated haptoglobin (HP), a positive acute phase protein capable of binding haemoglobin and removing it from the circulation to prevent iron loss, renal damage and inhibit microbe iron uptake, thus reducing its function as an antioxidant. Although, High HP levels have been reported in the blood of cattle with infections/diseases like mastitis, metritis, traumatic reticulitis, bacterial nephritis and bovine respiratory syncytial virus [16] and many others, there is no literature indicating its involvement in BVDV infection. Therefore, our finding seems to be unexpected, and to investigate the meaning of these two observations, further studies are needed.

Interestingly, among 69 proteins that have been altered by both biotypes only two proteins, integrin alpha 2b (ITGA2B) and integrin beta 3 (ITGB3), were differentially altered by cp and ncp BVDV biotypes. Integrins are the main cell surface receptors for proteins within the extracellular matrix (ECM); they enable cells to migrate, form strong adhesive junctions, and respond to ECM contact by differentiating and/or proliferating [18,19]. Our results indicate that 24h ncp BVDV infection decreased the levels of ITGA2B and ITGB3, whereas cp BVDV biotype significantly increased their expression levels. Both integrins are involved in integrin signalling pathway, one of the top ten pathways affected by ncp BVDV-infection. Protein alpha 6 (ITGA6) that was also down-regulated by ncp BVDV, is known to be a member of the integrin family involved in integrin signalling pathway, and was recently shown to be involved in cell differentiation [20]. This finding indicates that ncp BVDV unlike the cp counterpart, inhibited the level of communication of the ECM and cell differentiation. Finally, all the integrins affected by BVDV are also involved in caveolae-mediated endocytosis signalling pathway which was one of the top ten pathways affected by both ncp and cp BVDV biotypes.

In general, the observed effects of cp BVDV in this study are in agreement with our previous reports suggesting that cp BVDV, while promoting the expression of proteins involved in monocyte activation and differentiation, is inhibiting their antigen presentation to immunocompetent T cells, thus resulting in the uncontrolled inflammation, enhanced viral spread, and impaired anti-viral defense mechanisms in the host. Unlike the cp BVDV biotype, ncp BVDV increased the expression of proteins involved in compensatory survival and inhibition of cell activation mechanisms, promoting virus persistence [9,11].

## Conclusions

In this study, we identified bovine proteins whose expression altered significantly during BVDV infection compared to the uninfected monocytes. Those monocyte protein profiles distinguished between the two biotypes showed that cp BVDV had more profound effect on the protein expression levels with significantly increased number of down-regulated proteins and decreased number of up-regulated proteins compared to the ncp BVDV biotype. The use of GO showed profound biotype-related differences in all GO functional groups, indicating that low doses of BVDV infection can be crucial to understand the complex pathogenesis of BVDV infection. Also, systems biology network modelling identified multiple biotype-related differences in significant biological pathways that could explain the observed biological differences. In particular, our data indicated that only cp BVDV significantly increased the protein expression levels of Mx protein that is believed to be induced exclusively via signalling through the type I INF receptor. INF receptor signalling activates the adaptive immune responses, and suppression of this signal may be essential for the establishment of persistent infection that could explain the observed biological differences.

In this study, acute phase pathway was demonstrated to be the first significant pathway in both ncp and cp BVDV infection. Although, ncp and cp viruses altered different numbers of proteins in general, they had the same effects on the monocyte protein expression levels. Our finding indicates that ncp BVDV unlike the cp counterpart, inhibited the level of communication of the ECM and cell differentiation thus promoting the establishment of persistent infection. The differences in the expression of the integrins can also mean that cp BVDV infection induces monocytes to differentiate into macrophages, or, alternatively, that monocytes that have already embarked on the differentiation into macrophages, are more susceptible to cp BVDV infection.

Taken together, the combined use of GO information and systems biology network modelling extended our knowledge of the roles of ncp and cp BVDV biotypes in the production of persistent infection and cytopathic effects respectively.

## Methods

### Animals

Nine conventionally reared, healthy BVDV-free cows from a Holstein herd at the Mississippi State University Dairy Facility were used. The animals have been subjected to a comprehensive vaccination program, including Frontier 4 Plus Vaccine (IBR, BVD, PI3, RSV, Diamond Animal H, Inc). All animal used was approved by The Mississippi State University Institutional Animal Care and Use Committee. Peripheral blood mononuclear cells (PBMC) separated from the animals used in our study were tested for the expression of BVDV E2 transcripts with E2 BVDV specific primers by RT-PCR [8]. As we expected, all animals were BVDV mRNA-free (data not shown).

### Cell preparation

Blood samples (150 ml) were collected into Blood Collection Tubes (16×100 mm, Tyco Healthcare) by jugular venipuncture. Bovine PBMC were separated as described elsewhere [8,21]. Briefly, PBMC were isolated using Histopaque gradients (1.077 g/ml, Amersham Biosciences) and resuspended in RPMI-1640 supplemented with 10% FBS, 1% Glutamax-1 (Invitrogen), 5×10– 5 M 2-mercaptoethanol and 100 IU/ml Gentamicin (Invitrogen). Monocytes were separated from PBMC as described elsewhere [22]. Briefly, 40 ml of PBMC suspension (5×108 cells) was added to Petri-dish (150×25 mm, BD sciences) for 2 h at 37°C. Non-adherent cells were removed and the adherent cells were washed twice in PBS (Invitrogen). The yield of adherent cells was 20–30% of total PBMC number. After removing non-adherent populations (mostly T and B cells), adherent cells were incubated with mAbs to CD14 (MM61A, VMRD) followed by the addition of magnetic beads conjugated with mouse anti-IgG1 (Miltenyi Biotech, Auburn, CA) [21].CD14+ monocytes were positively selected by using magnetic cell separation technique according to the manufacturer’s instructions (Miltenyi Biotech). The final yield of bovine monocytes was 2–3% of total PBMC number.

### BVDV stocks and infection

BVDV biotypes were prepared as described elsewhere [12]. Briefly, the NADL (cp) biotype of BVDV was obtained from the American Type Culture Collection (ATCC) and amplified by growth in the bovine turbinate (BT) cell line (ATCC) according to the manufacturer’s handling procedures. For infection of BT cells, virus dilutions were made in DMEM with 4 mM L-glutamine, 4.5 g/l glucose, 1.5 g/l sodium bicarbonate and 10% horse serum. To measure the infectivity of the NADL biotype, the quantal method of Reed and Muench was performed and the tissue culture infectious dose 50 (TCID50) was determined. For the ncp BVDV biotype NY, we used the TCID50 suggested by the manufacturer (ATCC). To select the dose of cp BVDV that did not have a cytopathic effect on monocytes cultured for 48 h we assessed the viability of the infected cells by using trypan blue and light microscopy. BVDV biotype NADL at the multiplicity of infection (MOI) 0.002 had not affected the viability of bovine monocytes after 48 h of infection (data not shown). 5×106 monocytes were added to each well of a 6 well tissue culture plate and infected with cp and ncp BVDV biotypes at the same MOI of 0.002 for 24 h. After infection, at least 107 cells were pooled in one tube. All data were determined using triplicate monocyte cultures.

### Protein extraction by DDF

Differential detergent fractionation (DDF) sequentially extracts proteins using a series of detergents with increasing ionic strength, leading to an increase in proteome coverage. Proteins were isolated using DDF as previously described [23,24]. Briefly, cytosolic proteins were isolated and depleted by repeated washes in digitonin buffer. After the digitonin washes, proteins were sequentially extracted using triton X-100 (TX), deoxycholate (DOC), tween 40, and SDS buffers, respectively. To evaluate the quality of isolated proteins, 1% of the protein samples were compared using 10% SDS-PAGE (data not shown). For each of the detergent fractions, equal amounts of protein were precipitated with 25% trichloroacetic acid to remove salts and detergents. Protein pellets were solubilized and then digested with 100 ng of trypsin (50:1 ratio of substrate to enzyme) overnight at 37°C. Peptides were desalted using a peptide microtrap (Michrom BioResources, Inc.) and eluted by a 0.1% trifluoroacetic acid, 95% acetonitrile solution. Desalted peptides were dried and resuspended in 0.1% formic acid.

### 2D-LC ESI MS^2^

Proteomic analysis was carried out with duplicate samples of untreated, cp and ncp-BVDV infected bovine monocytes using 2D-LC ESI MS^2^ as described elsewhere [23,24]. Briefly, LC analysis was accomplished by strong cation exchange (SCX) followed by reverse phase (RP) liquid chromatography (LC) coupled directly in line with electrospray (ESI) ion trap MS. Each DDF fraction samples from three different infections were loaded into a LC gradient ion exchange system including a Thermo Separations P4000 quaternary gradient pump (ThermoElectron Corporation) coupled with a 0.32×100 mm BioBasic SCX column and run three times. A flow rate of 3 μl/minwas used for both SCX and RP columns. A salt gradient was applied in steps of 0, 5, 10, 15, 20, 25, 30, 35, 40, 45, 50, 57, 64, 71, 79, 90, 110, 300, and 700 mM ammonium acetate in 5% acetonitrile, 0.1% formic acid and the resultant peptides were loaded directly into the sample loop of a 0.18×100 mm BioBasic C18 RP LC column of a Proteome X workstation (ThermoElectron). The RP gradient used 0.1% formic acid in acetonitrile and increased the acetonitrile concentration in a linear gradient from 5% to 30% in 30 min and then 30% to 65% in 9 min followed by 95% for 5 min and 5% for 15 min.

The spectrum collection time was 59 min for every SCX step. The LCQ Deca ion trap mass spectrometer (ThermoElectron) was configured to optimize the duty cycle length with the quality of data acquired by alternating between a single full MS scan followed by three tandem MS scans on the three most intense precursor masses from full scan. The collision energy was normalized to 35%. Dynamic mass exclusion windows were set at 2 min, and all of the spectra were measured with an overall mass/charge (m/z) ration range of 200–2000.

### Protein identification and differential protein expression

Proteins were identified and analyzed as previously described [12]. All searches were done using TurboSEQUEST™ (Bioworks Browser 3.2; ThermoElectron) [25]. Mass spectra and tandem mass spectra were searched against an in silico trypsin-digested non-redundant protein database of Bos taurus downloaded from National Center for Biotechnology Institute (NCBI). Cysteine carboxyamidomethylation and methionine single and double oxidation were included in the search criteria. Decoy searches from a randomized version of the bovine protein database were conducted with tandem mass spectra as described above. The probability for peptide identification was estimated using a method described for Sequest data analysis and was set at p < 0.05 [26]. Probabilities of protein identifications being incorrect were calculated using published methods [27,28]. Differential protein expression analysis based on ΣXcorr was carried out as described in ProtQuant [29]. To correct for multiple testing, we determined the false discovery rate (FDR) for p value using published methods [30].

### Gene Ontology Annotation

Gene ontology (GO) analysis was carried using AgBase tools [31] to identify the molecular functions, biological processes and cellular component represented in our protein datasets. GORetriver tool was used to obtain all pre-existing GO annotations available for known proteins in our datasets. In addition, we used GOanna to provide additional GO annotation (i.e. predicted based on sequence orthologes and analysis of functional domains) for bovine proteins without existing annotation. All GO annotations for our datasets were grouped into more generalized categories using GOSlimViewer and summarized using the GOA and Whole Proteome GOSlim set. Subcategories in each of the three GOSlim functional categories are shown as a fold change between the percentages of GO terms identified in cp to those of ncp BVDV-infected monocytes in Figure 5.

### Modelling using Ingenuity Pathway Analysis

To visualize and explore networks which are significantly represented in our proteomic datasets we used Ingenuity Pathways Analysis (IPA; Ingenuity system, California). Only differentially expressed host proteins in BVDV-infected monocytes were analyzed by IPA. Each gene identifier was mapped to its corresponding gene object in the Ingenuity Pathways Knowledge Base (IPKB). IPKB selects "focus genes" to be used for generating biological networks and computes a score for each network from P-value which indicates the likelihood of the focus genes in a network being found together due to chance. We selected networks scoring ≥ 2, which have > 99% confidence of not being generated by chance [32,33]. Biological functions are assigned to each network by using annotations from scientific literature and stored in the IPKB. Fisher exact test is used to calculate the P-value determining the probability of each biological function/disease or pathway being assigned by chance. We used immunological cells as a filter and a *P*-value ≤ 0.05 to select highly significant functions/disease and pathways represented in our proteomic datasets.

## Competing interests

The authors declare that they have no competing interests.

## Authors' contributions

MA performed the data generation, analyzed and interpreted proteomic data and wrote the draft of the manuscript. FM, BN and LP helped to draft the manuscript. FM co-ordinated the project and assisted with functional analysis. BN participated in analyzing the proteomics data and assisted with system biology modelling. LP initiated the project and helped in interpretation of data. Study was part of MA graduate work. LP is the corresponding author and the major professor of MA. All authors read and approved the final manuscript.

## Supplementary Material

Additional file 1 The file is a list of proteins identified by DDF-MudPIT which are significantly altered by ncp BVDV infection compared to uninfected moncytes. File contains GenBank accession, symbol and description (name from NCBI). For each protein we provided the information about number of peptides, Sequest cross correlation (ΣXcorr) and the type of regulation. Click here for file

Additional file 2 The file is a list of proteins identified by DDF-MudPIT which are significantly altered by cp BVDV infection compared to uninfected moncytes. File contains GenBank accession, symbol and description (name from NCBI). For each protein we provided the information about number of peptides, Sequest cross correlation (ΣXcorr) and the type of regulation. Click here for file

## References

[B1] Kobrak A Weber EL [Bovine diarrhea virus: an update] Rev Argent Microbiol 1997 29 1 47 61 9229725

[B2] Houe H Epidemiological features and economical importance of bovine virus diarrhoea virus (BVDV) infections. Vet Microbiol 1999 64 2-3 89 107 10.1016/S0378-1135(98)00262-4 10028165

[B3] Hamers C Dehan P Couvreur B Letellier C Kerkhofs P Pastoret PP Diversity among bovine pestiviruses. Vet J 2001 161 2 112 122 10.1053/tvjl.2000.0504 11243683

[B4] Fulton RW Ridpath JF Ore S Confer AW Saliki JT Burge LJ Payton ME Bovine viral diarrhoea virus (BVDV) subgenotypes in diagnostic laboratory accessions: distribution of BVDV1a, 1b, and 2a subgenotypes. Vet Microbiol 2005 111 1-2 35 40 10.1016/j.vetmic.2005.10.002 16263224

[B5] Brock KV The persistence of bovine viral diarrhea virus. Biologicals 2003 31 2 133 135 10.1016/S1045-1056(03)00029-0 12770545

[B6] Brownlie J Clarke MC Howard CJ Pocock DH Pathogenesis and epidemiology of bovine virus diarrhoea virus infection of cattle. Ann Rech Vet 1987 18 2 157 166 3619343

[B7] Brock KV The many faces of bovine viral diarrhea virus. Vet Clin North Am Food Anim Pract 2004 20 1 1 3 10.1016/j.cvfa.2003.12.002 15062470

[B8] Boyd BL Lee TM Kruger EF Pinchuk LM Cytopathic and non-cytopathic bovine viral diarrhoea virus biotypes affect fluid phase uptake and mannose receptor-mediated endocytosis in bovine monocytes. Vet Immunol Immunopathol 2004 102 1-2 53 65 10.1016/j.vetimm.2004.06.009 15451615

[B9] Lee SR Nanduri B Pharr GT Stokes JV Pinchuk LM Bovine viral diarrhea virus infection affects the expression of proteins related to professional antigen presentation in bovine monocytes. Biochim Biophys Acta 2009 1794 1 14 22 18930168 10.1016/j.bbapap.2008.09.005

[B10] Franchini M Schweizer M Matzener P Magkouras I Sauter KS Mirkovitch J Peterhans E Jungi TW Evidence for dissociation of TLR mRNA expression and TLR agonist-mediated functions in bovine macrophages. Vet Immunol Immunopathol 2006 110 1-2 37 49 10.1016/j.vetimm.2005.09.002 16216336

[B11] Pinchuk GV Lee SR Nanduri B Honsinger KL Stokes JV Pinchuk LM Bovine viral diarrhea viruses differentially alter the expression of the protein kinases and related proteins affecting the development of infection and anti-viral mechanisms in bovine monocytes. Biochim Biophys Acta 2008 1784 9 1234 1247 18570900 10.1016/j.bbapap.2008.05.004

[B12] Pendarvis K Kumar R Burgess SC Nanduri B An automated proteomic data analysis workflow for mass spectrometry. BMC Bioinformatics 2009 10 Suppl 11 S17 10.1186/1471-2105-10-S11-S17 19811682 PMC3226188

[B13] Peterhans E Jungi TW Schweizer M BVDV and innate immunity. Biologicals 2003 31 2 107 112 10.1016/S1045-1056(03)00024-1 12770540

[B14] von Wussow P Jakschies D Hochkeppel HK Fibich C Penner L Deicher H The human intracellular Mx-homologous protein is specifically induced by type I interferons. Eur J Immunol 1990 20 9 2015 2019 10.1002/eji.1830200920 2120071

[B15] Baigent SJ Zhang G Fray MD Flick-Smith H Goodbourn S McCauley JW Inhibition of beta interferon transcription by noncytopathogenic bovine viral diarrhea virus is through an interferon regulatory factor 3-dependent mechanism. J Virol 2002 76 18 8979 8988 10.1128/JVI.76.18.8979-8988.2002 12186882 PMC136435

[B16] Heegaard PM Godson DL Toussaint MJ Tjornehoj K Larsen LE Viuff B Ronsholt L The acute phase response of haptoglobin and serum amyloid A (SAA) in cattle undergoing experimental infection with bovine respiratory syncytial virus. Vet Immunol Immunopathol 2000 77 1-2 151 159 10.1016/S0165-2427(00)00226-9 11068073 PMC7119828

[B17] Drakesmith H Prentice A Viral infection and iron metabolism. Nat Rev Microbiol 2008 6 7 541 552 10.1038/nrmicro1930 18552864

[B18] Delon I Brown NH Integrins and the actin cytoskeleton. Curr Opin Cell Biol 2007 19 1 43 50 10.1016/j.ceb.2006.12.013 17184985

[B19] Le Clainche C Carlier MF Regulation of actin assembly associated with protrusion and adhesion in cell migration. Physiol Rev 2008 88 2 489 513 10.1152/physrev.00021.2007 18391171

[B20] Meighan CM Schwarzbauer JE Temporal and spatial regulation of integrins during development. Curr Opin Cell Biol 2008 20 5 520 524 10.1016/j.ceb.2008.05.010 18603422 PMC2572561

[B21] Lee SR Pharr GT Boyd BL Pinchuk LM Bovine viral diarrhea viruses modulate toll-like receptors, cytokines and co-stimulatory molecules genes expression in bovine peripheral blood monocytes. Comp Immunol Microbiol Infect Dis 2008 31 5 403 418 10.1016/j.cimid.2007.06.006 17706777

[B22] Kruger EF Boyd BL Pinchuk LM Bovine monocytes induce immunoglobulin production in peripheral blood B lymphocytes. Dev Comp Immunol 2003 27 10 889 897 10.1016/S0145-305X(03)00080-6 12880638

[B23] Lee SR Pharr GT Cooksey AM McCarthy FM Boyd BL Pinchuk LM Differential detergent fractionation for non-electrophoretic bovine peripheral blood monocyte proteomics reveals proteins involved in professional antigen presentation. Dev Comp Immunol 2006 30 11 1070 1083 10.1016/j.dci.2006.02.002 16566999

[B24] McCarthy FM Burgess SC van den Berg BH Koter MD Pharr GT Differential detergent fractionation for non-electrophoretic eukaryote cell proteomics. J Proteome Res 2005 4 2 316 324 10.1021/pr049842d 15822906

[B25] Eng JK McCormack AL Yates JR An approach to correlate tandem mass spectral data of peptides with amino acid sequences in a protein database. J Am Soc Mass Spectrom 1994 5 976 989 10.1016/1044-0305(94)80016-2 24226387

[B26] Qian WJ Liu T Monroe ME Strittmatter EF Jacobs JM Kangas LJ Petritis K Camp DG Smith RD 2nd Probability-based evaluation of peptide and protein identifications from tandem mass spectrometry and SEQUEST analysis: the human proteome. J Proteome Res 2005 4 1 53 62 10.1021/pr0498638 15707357

[B27] Lopez-Ferrer D Martinez-Bartolome S Villar M Campillos M Martin-Maroto F Vazquez J Statistical model for large-scale peptide identification in databases from tandem mass spectra using SEQUEST. Anal Chem 2004 76 23 6853 6860 10.1021/ac049305c 15571333

[B28] MacCoss MJ Wu CC Yates JR 3rd Probability-based validation of protein identifications using a modified SEQUEST algorithm. Anal Chem 2002 74 21 5593 5599 10.1021/ac025826t 12433093

[B29] Bridges SM Magee GB Wang N Williams WP Burgess SC Nanduri B ProtQuant: a tool for the label-free quantification of MudPIT proteomics data. BMC Bioinformatics 2007 8 Suppl 7 S24 10.1186/1471-2105-8-S7-S24 18047724 PMC2099493

[B30] Benjamini Y Hochberg Y Controlling the false discovery rate: a practical and powerful approach to multiple testing. Journal of the Royal Statistical Society: Series B (Statistical Methodology) 1995 57 289 300

[B31] McCarthy FM Bridges SM Wang N Magee GB Williams WP Luthe DS Burgess SC AgBase: a unified resource for functional analysis in agriculture. Nucleic Acids Res 2007 35 Database issue D599 603 10.1093/nar/gkl936 17135208 PMC1751552

[B32] Gerling IC Singh S Lenchik NI Marshall DR Wu J New data analysis and mining approaches identify unique proteome and transcriptome markers of susceptibility to autoimmune diabetes. Mol Cell Proteomics 2006 5 2 293 305 16227630 10.1074/mcp.M500197-MCP200

[B33] Peddinti D Nanduri B Kaya A Feugang JM Burgess SC Memili E Comprehensive proteomic analysis of bovine spermatozoa of varying fertility rates and identification of biomarkers associated with fertility. BMC Syst Biol 2008 2 19 10.1186/1752-0509-2-19 18294385 PMC2291030

