# Supplementary material for: Analysis of Bovine Viral Diarrhea Viruses-infected monocytes: identification of cytopathic and non-cytopathic biotype differences
Source: BMC Bioinformatics. 2010 Oct 7;11(Suppl 6):S9. doi: 10.1186/1471-2105-11-S6-S9 (PMC3026383; doi:10.1186/1471-2105-11-S6-S9)
Supplement: Additional file 1 — The file is a list of proteins identified by DDF-MudPIT which are significantly altered by ncp BVDV infection compared to uninfected moncytes. File contains GenBank accession, symbol and description (name from NCBI). For each protein we provided the information about number of peptides, Sequest cross correlation (ΣXcorr) and the type of regulation. [file 1471-2105-11-S6-S9-S1.pdf]

GenBank Accession	Symbol	Description	Peptides		Σxcorr		Regulation
			Control	Ncp	Control	Ncp	
NP_001029595	PSMD7	26S proteasome non-ATPase regulatory subunit 7	4	0	13.29	0.00	Down
NP_001029382	PAPSS1	3 -phosphoadenosine 5 -phosphosulfate synthase 1	21	11	68.72	36.93	Down
NP_001069017	ACAT2	acetyl-Coenzyme A acetyltransferase 2	0	4	0.00	12.68	Up
NP_776651	ACTR3	actin-related protein 3	3	13	8.43	50.57	Up
NP_001030240	ACSF3	acyl-CoA synthetase family member 3, mitochondrial precursor	3	0	9.03	0.00	Down
NP_001033131	ACSL6	acyl-CoA synthetase long-chain family member 6	30	44	94.68	152.68	Up
NP_777171	ACSS1	acyl-CoA synthetase short-chain family member 1	9	2	32.26	8.22	Down
NP_776314	AK2	adenylate kinase 2	42	26	143.21	90.45	Down
NP_001030182	CAP1	adenylyl cyclase-associated protein 1	1	7	2.58	29.15	Up
NP_776307	SERPINA1	alpha-1-antiproteinase precursor	0	3	0.00	8.52	Up
NP_776409	AHSG	alpha-2-HS-glycoprotein precursor	0	28	0.00	112.85	Up
NP_001103265	A2M	alpha-2-macroglobulin	5	27	13.42	97.15	Up
NP_777141	ANXA2	annexin A2	6	27	17.47	89.23	Up
NP_001030402	ANXA3	annexin A3	0	4	0.00	13.38	Up
NP_001070294	ANTXR2	anthrax toxin receptor 2	0	3	0.00	14.71	Up
NP_001029870	SERPINC1	antithrombin-III precursor	0	3	0.00	9.88	Up
NP_001157408	APOBEC3A	apolipoprotein B mRNA editing enzyme, catalytic polypeptide-like 3A	0	3	0.00	9.03	Up
NP_001017942	ARG2	arginase 2 precursor	0	36	0.00	139.61	Up
NP_001092448	ATL3	atlastin 3	30	19	116.56	74.86	Down
NP_776929	ATP6V1A	ATPase, H+ transporting, lysosomal V1 subunit A	5	13	12.75	36.88	Up
NP_001099121	BOLA-N	BOLA class I histocompatibility antigen, alpha chain BL3-6 precursor	1	7	3.82	20.88	Up
NP_001073106	CTNNA3	catenin, alpha 3	3	0	6.79	0.00	Down
NP_001030403	CTSA	cathepsin A precursor	0	4	0.00	13.96	Up
NP_001159993	CTSD	cathepsin D	1	8	2.20	25.02	Up
NP_001028787	CTSS	cathepsin S precursor	1	9	3.07	28.76	Up
NP_776777	IGF2R	cation-independent mannose-6-phosphate receptor	2	7	4.01	21.77	Up
NP_001156885	CD163	CD163 molecule	0	13	0.00	43.99	Up
NP_001039367	CD68	CD68 molecule	1	14	2.38	50.95	Up
NP_776304	F5	coagulation factor V precursor	0	5	0.00	14.82	Up
NP_001068871	CSF1R	colony stimulating factor 1 receptor	0	5	0.00	16.09	Up
NP_777225	CFDP2	craniofacial development protein 2	9	2	27.54	7.38	Down

NP_001073117	CRIP2	cysteine-rich protein 2	3	0	13.89	0.00	Down
NP_001030467	POR	cytochrome P450 reductase	22	49	75.74	174.98	Up
NP_001095800	DCP1A	DCP1 decapping enzyme homolog A	0	3	0.00	10.07	Up
NP_001068758	MCM5	DNA replication licensing factor MCM5	6	2	19.06	4.29	Down
NP_001069922	ELAVL1	ELAV (embryonic lethal, abnormal vision, Drosophila)-like 1 (Hu antigen R)	1	5	3.93	19.30	Up
NP_001070598	ETFDH	electron-transferring-flavoprotein dehydrogenase precursor	0	3	0.00	8.66	Up
NP_001039595	SH3GL1	endophilin-A2	9	4	23.66	9.85	Down
NP_851352	ECE1	endothelin converting enzyme 1	0	4	0.00	12.26	Up
NP_001068589	EEF2	eukaryotic translation elongation factor 2	0	5	0.00	19.11	Up
NP_001071411	ESYT1	extended synaptotagmin-like protein 1	111	72	375.84	235.57	Down
NP_001136389	FGB	fibrinogen beta chain	0	4	0.00	14.46	Up
NP_001095385	ALDOA	fructose-bisphosphate aldolase A	11	22	39.32	79.68	Up
NP_001095811	LGALS3	galectin 3	2	9	6.11	28.19	Up
NP_001092461	FGR	Gardner-Rasheed feline sarcoma viral (v-fgr) oncogene homolog	10	0	38.97	0.00	Down
NP_001035560	HP	haptoglobin precursor	3	0	8.13	0.00	Down
NP_001033764	TRAP1	heat shock protein 75 kDa, mitochondrial precursor	4	0	14.73	0.00	Down
NP_001014912	HMOX1	heme oxygenase (decyclizing) 1	2	25	4.97	80.14	Up
NP_001070368	HNRPLL	heterogeneous nuclear ribonucleoprotein L-like	3	0	9.07	0.00	Down
NP_001070388	HNRNPU	heterogeneous nuclear ribonucleoprotein U	31	18	98.49	58.35	Down
NP_001095399	HK3	hexokinase 3	6	1	17.27	2.97	Down
NP_001098910	HAL	histidine ammonia-lyase	0	3	0.00	9.12	Up
NP_001075211	HIST1H2BN	histone cluster 1, H2bn	13	30	40.74	98.13	Up
NP_001073755	MGC143117	hypothetical protein LOC522040	0	3	0.00	7.16	Up
NP_001071555	C19H17orf62	hypothetical protein LOC618343	4	0	13.38	0.00	Down
NP_001014929	ITGA2B	integrin alpha 2b	26	12	111.92	50.96	Down
NP_001035046	ITGAM	integrin alpha M	13	21	31.80	57.89	Up
NP_001103451	ITGA6	integrin, alpha 6	3	0	9.97	0.00	Down
NP_001095675	KTN1	kinectin 1	6	0	14.94	0.00	Down
NP_001096765	LMNB1	lamin B1	10	3	34.72	9.15	Down
NP_776526	LGMN	legumain precursor	0	9	0.00	30.45	Up
NP_001039929	MPEG1	macrophage expressed gene 1	3	13	8.57	39.67	Up
NP_776986	MAN2B1	mannosidase, alpha, class 2B, member 1	0	3	0.00	7.05	Up
NP_001069640	MARCKSL1	MARCKS-related protein	0	5	0.00	19.19	Up
NP_777169	MMP9	matrix metalloproteinase-9 precursor	0	17	0.00	53.75	Up
NP_001069180	MTA2	metastasis associated 1 family, member 2	4	0	10.12	0.00	Down

NP_001137332	MACF1	microtubule-actin crosslinking factor 1	0	3	0.00	7.69	Up
NP_001068626	MCM4	minichromosome maintenance complex component 4	19	5	59.77	18.15	Down
NP_001028783	UCP2	mitochondrial uncoupling protein 2	1	6	2.97	19.17	Up
NP_776433	CD14	monocyte differentiation antigen CD14 precursor	1	10	2.98	31.87	Up
NP_001069744	MARCKS	myristoylated alanine-rich C-kinase substrate	0	7	0.00	26.44	Up
NP_788833	NDUFB7	NADH dehydrogenase	0	3	0.00	10.22	Up
NP_777183	NPC1	Niemann-Pick disease, type C1	0	3	0.00	7.52	Up
NP_776683	CALD1	non-muscle caldesmon	4	0	12.03	0.00	Down
NP_001068667	NUCB1	nucleobindin-1 precursor	10	3	30.51	7.85	Down
NP_001029412	OAT	ornithine aminotransferase precursor	22	58	72.52	216.37	Up
NP_776856	PRDX1	peroxiredoxin 1	2	11	7.48	46.13	Up
NP_777174	PRDX5	peroxiredoxin 5 precursor	7	16	20.35	49.69	Up
NP_001095447	PICALM	phosphatidylinositol-binding clathrin assembly protein	1	6	2.74	17.47	Up
NP_777176	PARP1	poly (ADP-ribose) polymerase family, member 1	0	3	0.00	7.95	Up
NP_803485	GALNT1	polypeptide N-acetylgalactosaminyltransferase 1	0	4	0.00	12.86	Up
XP_590771	CTBP1	PREDICTED: C-terminal binding protein 1	5	1	21.13	2.57	Down
XP_614269	FLNA	PREDICTED: filamin A, alpha (actin binding protein 280)	42	63	127.16	212.80	Up
XP_615814	FKBP5	PREDICTED: FK506 binding protein 5	3	0	9.41	0.00	Down
XP_616376	ITGB3	PREDICTED: integrin, beta 3 (platelet glycoprotein IIIa, antigen CD61) isoform 1	20	6	61.70	21.27	Down
XP_614626	NCL	PREDICTED: nucleolin isoform 1	23	5	69.56	14.17	Down
XP_581432	SERPINB2	PREDICTED: plasminogen activator inhibitor-2 isoform 1	0	9	0.00	30.63	Up
XP_599431	PTPRC	PREDICTED: protein tyrosine phosphatase, receptor type, C	17	29	67.06	100.01	Up
XP_590109	PKM2	PREDICTED: pyruvate kinase, muscle	39	58	146.45	205.43	Up
XP_001255510	LPCAT1	PREDICTED: similar to 1-acylglycerophosphocholine O-acyltransferase 1 (Lung-type acyl-CoA:lysophosphatidylcholine acyltransferase 1) (Lysophosphatidylcholine acyltransferase 1) (Acyltransferase-like 2) (Phosphonoformate immuno-associated protein 3)	0	3	0.00	7.55	Up
XP_581045	LOC504861	PREDICTED: similar to cationic amino acid transporter 5	0	7	0.00	23.35	Up
XP_001789660	ARAP1	PREDICTED: similar to centaurin delta 2	0	3	0.00	11.14	Up
XP_871851	HTT	PREDICTED: similar to huntingtin	0	4	0.00	9.15	Up
XP_869445	MCM2	PREDICTED: similar to KIAA0030 isoform 3, partial	12	3	44.42	10.11	Down
XP_001787859	LRPPRC	PREDICTED: similar to Leucine-rich PPR motif-containing protein, mitochondrial precursor (130 kDa leucine-rich protein) (LRP 130) (GP130)	12	4	38.93	11.89	Down
XP_877405	LUC7L2	PREDICTED: similar to LUC7-like 2 isoform 2	3	0	7.21	0.00	Down

XP_001253929	LOC786695	PREDICTED: similar to major histocompatibility complex, class II, DR beta 3	0	4	0.00	14.64	Up
XP_580330	ARHGAP17	PREDICTED: similar to nadrin	5	1	29.06	6.05	Down
XP_001257022	LOC790592	PREDICTED: similar to Neuroblast differentiation-associated protein AHNAK (Desmoyokin), partial	6	16	20.08	54.62	Up
XP_874112	MYCN	PREDICTED: similar to N-myc protein	4	0	7.57	0.00	Down
XP_589182	PHKB	PREDICTED: similar to Phosphorylase b kinase regulatory subunit beta (Phosphorylase kinase subunit beta)	3	0	5.56	0.00	Down
XP_581459	EHD4	PREDICTED: similar to pincher isoform 1	0	5	0.00	18.84	Up
XP_873531	KCTD12	PREDICTED: similar to potassium channel tetramerization domain containing 12	0	7	0.00	23.52	Up
XP_001251163	IQGAP1	PREDICTED: similar to Ras GTPase-activating-like protein IQGAP1 (p195)	3	14	10.61	46.55	Up
XP_586753	SACS	PREDICTED: similar to Sacsin	0	3	0.00	6.33	Up
XP_611883	ALDH5A1	PREDICTED: similar to succinic semialdehyde dehydrogenase	0	4	0.00	13.16	Up
XP_001252942	TREML1	PREDICTED: similar to Trem-like transcript 1 protein precursor (TLT-1) (Triggering receptor expressed on myeloid cells-like protein 1)	8	1	28.19	3.05	Down
XP_613697	USP34	PREDICTED: similar to ubiquitin specific peptidase 34 isoform 1, partial	0	3	0.00	6.93	Up
XP_871340	UGGT1	PREDICTED: similar to UDP-glucose ceramide glucosyltransferase-like 1 isoform 2	41	27	140.35	83.94	Down
XP_001788407	YLPM1	PREDICTED: similar to YLP motif containing 1	3	0	7.92	0.00	Down
XP_588094	SPTBN1	PREDICTED: spectrin, beta, non-erythrocytic 1 isoform 1	23	8	96.24	37.21	Down
NP_001069119	PDXK	pyridoxal kinase	0	9	0.00	30.16	Up
NP_001068837	RANBP3	RAN binding protein 3	6	1	16.96	2.63	Down
NP_001096578	RAVER1	RAVER1	6	1	24.01	4.94	Down
NP_001095381	RCC2	regulator of chromosome condensation 2	15	8	47.97	22.40	Down
NP_001069329	SAMHD1	SAM domain and HD domain-containing protein 1	38	20	140.84	73.48	Down
NP_001068643	SEC11C	SEC11 homolog C	3	0	9.19	0.00	Down
NP_001069130	SDS	serine dehydratase	0	5	0.00	22.65	Up
NP_803450	TF	serotransferrin precursor	0	3	0.00	11.43	Up
NP_851335	ALB	serum albumin precursor	142	47	433.87	148.32	Down
NP_001096572	SPN	sialophorin	7	1	25.81	2.44	Down
NP_001069536	SLC9A9	solute carrier family 9 (sodium/hydrogen exchanger), member 9	3	0	13.82	0.00	Down
NP_001096741	SF3B2	splicing factor 3b, subunit 2, 145kDa	4	0	11.19	0.00	Down
NP_963285	SOD2	superoxide dismutase 2, mitochondrial precursor	50	365	201.86	1561.29	Up

NP_001069299	SNAP23	synaptosomal-associated protein 23	3	0	10.94	0.00	Down
NP_777050	TXNRD1	thioredoxin reductase 1	1	6	3.03	19.48	Up
NP_776621	THBS1	thrombospondin 1 precursor	5	17	18.66	60.13	Up
NP_001095318	TGM3	transglutaminase 3	0	31	0.00	114.27	Up
NP_001029788	TMEM183A	transmembrane protein 183A	3	0	6.20	0.00	Down
NP_001091527	TAP1	transporter 1, ATP-binding cassette, sub-family B	18	10	70.72	35.70	Down
NP_001137572	TUBB1	tubulin, beta 1	8	1	26.82	3.76	Down
NP_001020491	TWF1	twinfilin-1	0	5	0.00	16.40	Up
NP_776848	PLAUR	urokinase plasminogen activator surface receptor precursor	0	10	0.00	27.85	Up
NP_001039723	VPS35	vacuolar protein sorting-associated protein 35	1	5	1.45	13.70	Up
NP_001030222	VTN	vitronectin	0	5	0.00	18.08	Up
NP_776911	VDAC2	voltage-dependent anion-selective channel protein 2	19	12	65.04	40.01	Down
NP_001039811	WDR1	WD repeat-containing protein 1	7	17	28.15	65.48	Up
