# Supplementary material for: Analysis of Bovine Viral Diarrhea Viruses-infected monocytes: identification of cytopathic and non-cytopathic biotype differences
Source: BMC Bioinformatics. 2010 Oct 7;11(Suppl 6):S9. doi: 10.1186/1471-2105-11-S6-S9 (PMC3026383; doi:10.1186/1471-2105-11-S6-S9)
Supplement: Additional file 2 — The file is a list of proteins identified by DDF-MudPIT which are significantly altered by cp BVDV infection compared to uninfected moncytes. File contains GenBank accession, symbol and description (name from NCBI). For each protein we provided the information about number of peptides, Sequest cross correlation (ΣXcorr) and the type of regulation. [file 1471-2105-11-S6-S9-S2.pdf]

| GenBank Accession | Symbol | Description | Peptides | | Σxcorr | | Regulation |
|---|---|---|---|---|---|---|---|
| | | | Control | Cp | Control | Cp | |
| NP_001069498 | OGDH | 2-oxoglutarate dehydrogenase E1 component, mitochondrial precursor | 14 | 6 | 40.97 | 19.56 | Down |
| NP_001029382 | PAPSS1 | 3 -phosphoadenosine 5 -phosphosulfate synthase 1 | 21 | 11 | 68.72 | 34.01 | Down |
| NP_776759 | HSD17B10 | 3-hydroxyacyl-CoA dehydrogenase type-2 | 30 | 12 | 110.56 | 46.06 | Down |
| NP_001030419 | ACAA2 | 3-ketoacyl-CoA thiolase, mitochondrial | 35 | 19 | 132.12 | 73.53 | Down |
| NP_001028786 | RPS18 | 40S ribosomal protein S18 | 3 | 0 | 6.74 | 0.00 | Down |
| NP_858053 | ADAMTS4 | a disintegrin and metalloproteinase with thrombospondin motifs 4 precursor | 0 | 3 | 0.00 | 8.35 | Up |
| NP_001015606 | ABHD10 | abhydrolase domain containing 10 precursor | 3 | 0 | 9.80 | 0.00 | Down |
| NP_001039540 | ACAT1 | acetyl-Coenzyme A acetyltransferase 1 precursor | 21 | 9 | 68.47 | 29.74 | Down |
| NP_001029491 | ACAA1 | acetyl-Coenzyme A acyltransferase 1 | 6 | 2 | 22.57 | 5.23 | Down |
| NP_001029885 | ARPC2 | actin-related protein 2/3 complex subunit 2 | 3 | 9 | 9.04 | 25.44 | Up |
| NP_776651 | ACTR3 | actin-related protein 3 | 3 | 9 | 8.43 | 29.52 | Up |
| NP_001030240 | ACSF3 | acyl-CoA synthetase family member 3, mitochondrial precursor | 3 | 0 | 9.03 | 0.00 | Down |
| NP_777171 | ACSS1 | acyl-CoA synthetase short-chain family member 1 | 9 | 1 | 32.26 | 2.92 | Down |
| NP_001017933 | ACADSB | acyl-Coenzyme A dehydrogenase, short/branched chain | 4 | 1 | 17.35 | 2.29 | Down |
| NP_776314 | AK2 | adenylate kinase 2 | 42 | 18 | 143.21 | 66.67 | Down |
| NP_001030490 | APMAP | adipocyte plasma membrane-associated protein | 11 | 4 | 37.79 | 9.11 | Down |
| NP_001075907 | ADPGK | ADP-dependent glucokinase precursor | 5 | 1 | 15.77 | 1.64 | Down |
| NP_001071350 | C22H3ORF 64 | AER61 glycosyltransferase precursor | 3 | 0 | 9.79 | 0.00 | Down |
| NP_001099116 | ALDH4A1 | aldehyde dehydrogenase 4A1 precursor | 26 | 16 | 79.02 | 46.02 | Down |
| NP_001068835 | ALDH2 | aldehyde dehydrogenase, mitochondrial precursor | 164 | 132 | 543.53 | 455.47 | Down |
| NP_776307 | SERPINA1 | alpha-1-antiproteinase precursor | 0 | 7 | 0.00 | 17.93 | Up |
| NP_776409 | AHSG | alpha-2-HS-glycoprotein precursor | 0 | 65 | 0.00 | 255.74 | Up |
| NP_001103265 | A2M | alpha-2-macroglobulin | 5 | 17 | 13.42 | 56.35 | Up |
| NP_786978 | ANXA1 | annexin A1 | 28 | 40 | 87.12 | 136.37 | Up |
| NP_776667 | APOA1 | apolipoprotein A-I precursor | 1 | 11 | 2.99 | 31.96 | Up |
| NP_001017942 | ARG2 | arginase 2 precursor | 0 | 22 | 0.00 | 89.86 | Up |
| NP_001092448 | ATL3 | atlastin 3 | 30 | 21 | 116.56 | 83.49 | Down |
| NP_777109 | ATP5A1 | ATP synthase subunit alpha, mitochondrial precursor | 127 | 87 | 435.92 | 319.19 | Down |
| NP_001033590 | ATP5F1 | ATP synthase subunit b, mitochondrial precursor | 12 | 6 | 32.34 | 15.06 | Down |
| NP_788812 | ATP5I | ATP synthase subunit e, mitochondrial | 9 | 3 | 24.85 | 10.11 | Down |
| NP_001068604 | ATP5C1 | ATP synthase subunit gamma, mitochondrial precursor | 10 | 2 | 41.32 | 11.16 | Down |

| | | | | | | | |
|---|---|---|---|---|---|---|---|
| NP_776669 | ATP5O | ATP synthase subunit O, mitochondrial precursor | 34 | 19 | 110.93 | 56.89 | Down |
| NP_776929 | ATP6V1A | ATPase, H+ transporting, lysosomal V1 subunit A | 5 | 13 | 12.75 | 34.76 | Up |
| NP_776417 | APOH | beta-2-glycoprotein 1 precursor | 0 | 3 | 0.00 | 7.96 | Up |
| NP_001099121 | BOLA-N | BOLA class I histocompatibility antigen, alpha chain BL3-6 precursor | 1 | 6 | 3.82 | 20.22 | Up |
| NP_001029840 | BLA-DQB | BOLA class II histocompatibility antigen, DQB*0101 beta chain precursor | 5 | 1 | 23.13 | 2.95 | Down |
| NP_001076949 | CAPZA1 | capping protein (actin filament) muscle Z-line, alpha 1 | 5 | 10 | 16.82 | 37.95 | Up |
| NP_001030463 | CAT | catalase | 4 | 0 | 11.96 | 0.00 | Down |
| NP_001073106 | CTNNA3 | catenin, alpha 3 | 3 | 0 | 6.79 | 0.00 | Down |
| NP_001030403 | CTSA | cathepsin A precursor | 0 | 4 | 0.00 | 10.68 | Up |
| NP_786973 | M6PR | cation-dependent mannose-6-phosphate receptor precursor | 4 | 12 | 15.64 | 46.03 | Up |
| NP_001156885 | CD163 | CD163 molecule | 0 | 7 | 0.00 | 27.99 | Up |
| NP_001039367 | CD68 | CD68 molecule | 1 | 16 | 2.38 | 57.32 | Up |
| NP_001029907 | CD74 | CD74 antigen | 5 | 0 | 12.71 | 0.00 | Down |
| NP_776302 | F2 | coagulation factor II | 0 | 5 | 0.00 | 16.27 | Up |
| NP_776304 | F5 | coagulation factor V precursor | 0 | 41 | 0.00 | 151.18 | Up |
| NP_001073117 | CRIP2 | cysteine-rich protein 2 | 3 | 0 | 13.89 | 0.00 | Down |
| NP_001096720 | CYB5R3 | cytochrome b5 reductase 3 | 30 | 18 | 109.56 | 75.23 | Down |
| NP_001002891 | COX5A | cytochrome c oxidase subunit 5A, mitochondrial precursor | 10 | 5 | 33.72 | 16.48 | Down |
| YP_209208 | COX2 | cytochrome c oxidase subunit II | 13 | 6 | 51.92 | 22.32 | Down |
| NP_001030467 | POR | cytochrome P450 reductase | 22 | 35 | 75.74 | 115.68 | Up |
| NP_001076882 | CYP27A1 | cytochrome P450, family 27, subfamily A, polypeptide 1 | 13 | 3 | 48.19 | 9.83 | Down |
| NP_001068936 | DDX1 | DEAD (Asp-Glu-Ala-Asp) box polypeptide 1 | 3 | 0 | 9.67 | 0.00 | Down |
| NP_001039861 | DCI | dodecenoyl-Coenzyme A delta isomerase | 3 | 0 | 7.78 | 0.00 | Down |
| NP_001094543 | DDOST | dolichyl-diphosphooligosaccharide--protein glycosyltransferase 48 kDa subunit precursor | 18 | 10 | 71.67 | 40.47 | Down |
| NP_776632 | TUFM | elongation factor Tu, mitochondrial precursor | 11 | 3 | 35.81 | 11.28 | Down |
| NP_976237 | EMD | emerin | 5 | 2 | 17.14 | 6.09 | Down |
| NP_001071461 | SH3GLB1 | endophilin-B1 | 3 | 0 | 9.89 | 0.00 | Down |
| NP_001069096 | ERAP2 | endoplasmic reticulum aminopeptidase 2 | 22 | 6 | 75.78 | 17.79 | Down |
| NP_001030204 | ERP44 | endoplasmic reticulum resident protein ERp44 precursor | 26 | 18 | 98.18 | 72.33 | Down |
| NP_851352 | ECE1 | endothelin converting enzyme 1 | 0 | 4 | 0.00 | 13.16 | Up |
| NP_001096818 | ERO1L | ERO1-like precursor | 36 | 24 | 135.23 | 92.69 | Down |
| NP_001029635 | ES1 | es1 protein | 21 | 12 | 75.12 | 40.14 | Down |
| NP_001003658 | EIF5A | eukaryotic translation initiation factor 5A-1 | 3 | 0 | 9.85 | 0.00 | Down |
| NP_001071411 | ESYT1 | extended synaptotagmin-like protein 1 | 111 | 57 | 375.84 | 185.15 | Down |

| | | | | | | | |
|---|---|---|---|---|---|---|---|
| NP_776642 | EZR | ezrin | 9 | 3 | 27.06 | 10.08 | Down |
| NP_001070314 | FUBP1 | far upstream element-binding protein | 16 | 8 | 60.31 | 31.88 | Down |
| NP_001070488 | FAF2 | FAS-associated factor 2 | 3 | 0 | 13.86 | 0.00 | Down |
| NP_001136389 | FGB | fibrinogen beta chain | 0 | 9 | 0.00 | 31.07 | Up |
| NP_776336 | FGG | fibrinogen gamma-B chain precursor | 0 | 5 | 0.00 | 16.39 | Up |
| NP_001028798 | FGA | fibrinogen, alpha polypeptide precursor | 1 | 9 | 2.70 | 28.03 | Up |
| NP_001010996 | FCN2 | ficolin 2 precursor | 4 | 0 | 13.25 | 0.00 | Down |
| NP_001098884 | FYB | FYN binding protein | 4 | 0 | 12.02 | 0.00 | Down |
| NP_001095811 | LGALS3 | galectin 3 | 2 | 11 | 6.11 | 31.72 | Up |
| NP_001094721 | IFI30 | gamma-inducible protein 30 | 0 | 4 | 0.00 | 14.04 | Up |
| NP_001092461 | FGR | Gardner-Rasheed feline sarcoma viral (v-fgr) oncogene homolog | 10 | 1 | 38.97 | 2.48 | Down |
| NP_001071432 | GLS | glutaminase | 11 | 2 | 41.05 | 6.97 | Down |
| NP_001093766 | GPD2 | glycerol-3-phosphate dehydrogenase 2, mitochondrial precursor | 27 | 21 | 97.70 | 66.97 | Down |
| NP_001035560 | HP | haptoglobin precursor | 3 | 0 | 8.13 | 0.00 | Down |
| NP_001033764 | TRAP1 | heat shock protein 75 kDa, mitochondrial precursor | 4 | 0 | 14.73 | 0.00 | Down |
| NP_001014912 | HMOX1 | heme oxygenase (decyclizing) 1 | 2 | 20 | 4.97 | 66.66 | Up |
| NP_001035612 | HNRNPAB | heterogeneous nuclear ribonucleoprotein A/B | 14 | 5 | 50.86 | 14.51 | Down |
| NP_001070368 | HNRPLL | heterogeneous nuclear ribonucleoprotein L-like | 3 | 0 | 9.07 | 0.00 | Down |
| NP_776765 | HINT2 | histidine triad nucleotide-binding protein 2, mitochondrial precursor | 10 | 3 | 32.78 | 9.00 | Down |
| NP_001075211 | HIST1H2BN | histone cluster 1, H2bn | 13 | 31 | 40.74 | 104.52 | Up |
| NP_001039480 | IMMT | inner membrane protein, mitochondrial (mitofilin) | 5 | 1 | 19.93 | 2.91 | Down |
| NP_001039445 | STT3A | integral membrane protein 1 | 7 | 1 | 18.49 | 2.83 | Down |
| NP_001014929 | ITGA2B | integrin alpha 2b | 26 | 50 | 111.92 | 224.00 | Up |
| NP_776365 | MX1 | interferon-induced GTP-binding protein Mx1 | 0 | 3 | 0.00 | 11.99 | Up |
| NP_786984 | IDH2 | isocitrate dehydrogenase 2 (NADP+), mitochondrial precursor | 70 | 48 | 238.47 | 164.97 | Down |
| NP_777069 | IDH3A | isocitrate dehydrogenase 3 (NAD+) alpha precursor | 20 | 10 | 59.95 | 33.84 | Down |
| NP_001029554 | IVD | isovaleryl-CoA dehydrogenase, mitochondrial precursor | 20 | 13 | 74.23 | 50.50 | Down |
| NP_001095675 | KTN1 | kinectin 1 | 6 | 0 | 14.94 | 0.00 | Down |
| NP_001093799 | LBR | lamin B receptor | 9 | 3 | 23.64 | 10.35 | Down |
| NP_776526 | LGMN | legumain precursor | 0 | 4 | 0.00 | 12.40 | Up |
| NP_001015569 | LONP1 | lon protease homolog, mitochondrial precursor | 4 | 1 | 11.75 | 2.45 | Down |
| NP_788838 | SSB | lupus La protein homolog | 6 | 1 | 22.02 | 2.23 | Down |
| NP_001068592 | LAMP1 | lysosome-associated membrane glycoprotein 1 precursor | 5 | 17 | 10.39 | 40.62 | Up |
| NP_001039929 | MPEG1 | macrophage expressed gene 1 | 3 | 7 | 8.57 | 23.52 | Up |
| NP_001029800 | MDH1 | malate dehydrogenase, cytoplasmic | 5 | 1 | 21.16 | 5.03 | Down |
| NP_001068647 | MPDU1 | mannose-P-dolichol utilization defect 1 | 3 | 0 | 9.88 | 0.00 | Down |

| | | | | | | | |
|---|---|---|---|---|---|---|---|
| NP_001069640 | MARCKSL1 | MARCKS-related protein | 0 | 6 | 0.00 | 21.47 | Up |
| NP_001068626 | MCM4 | minichromosome maintenance complex component 4 | 19 | 4 | 59.77 | 11.97 | Down |
| NP_786990 | ATP5B | mitochondrial ATP synthase beta subunit precursor | 182 | 123 | 652.18 | 441.29 | Down |
| NP_776760 | HADHA | mitochondrial trifunctional protein, alpha subunit | 40 | 21 | 119.33 | 72.28 | Down |
| NP_851357 | MAOA | monoamine oxidase A | 13 | 4 | 38.50 | 16.27 | Down |
| NP_776433 | CD14 | monocyte differentiation antigen CD14 precursor | 1 | 9 | 2.98 | 27.76 | Up |
| NP_786974 | MYL6 | myosin light polypeptide 6 | 14 | 22 | 43.37 | 62.99 | Up |
| NP_777259 | MYH10 | myosin-10 | 10 | 17 | 32.56 | 59.75 | Up |
| NP_001069744 | MARCKS | myristoylated alanine-rich C-kinase substrate | 0 | 8 | 0.00 | 32.10 | Up |
| NP_787023 | NDUFA5 | NADH dehydrogenase ubiquinone] 1 alpha subcomplex subunit 5 | 3 | 0 | 9.56 | 0.00 | Down |
| NP_788833 | NDUFB7 | NADH dehydrogenase ubiquinone] 1 beta subcomplex subunit 7 | 0 | 4 | 0.00 | 12.32 | Up |
| NP_788815 | NDUFC2 | NADH dehydrogenase ubiquinone] 1 subunit C2 | 3 | 0 | 6.54 | 0.00 | Down |
| NP_786994 | NDUFS4 | NADH dehydrogenase ubiquinone] iron-sulfur protein 4, mitochondrial precursor | 4 | 0 | 10.71 | 0.00 | Down |
| NP_776683 | CALD1 | non-muscle caldesmon | 4 | 0 | 12.03 | 0.00 | Down |
| NP_001068667 | NUCB1 | nucleobindin-1 precursor | 10 | 4 | 30.51 | 11.59 | Down |
| NP_001029412 | OAT | ornithine aminotransferase precursor | 22 | 49 | 72.52 | 173.10 | Up |
| NP_001039499 | PTCD3 | pentatricopeptide repeat-containing protein 3, mitochondrial precursor | 3 | 0 | 9.08 | 0.00 | Down |
| NP_001039598 | PUF60 | poly(U)-binding-splicing factor PUF60 | 3 | 0 | 10.70 | 0.00 | Down |
| NP_776993 | PABPC1 | polyadenylate-binding protein 1 | 4 | 1 | 13.46 | 2.14 | Down |
| NP_776867 | PTBP1 | polypyrimidine tract-binding protein 1 | 32 | 20 | 112.58 | 68.66 | Down |
| XP_601044 | ABCA13 | PREDICTED: ATP-binding cassette, sub-family A (ABC1), member 13 | 0 | 3 | 0.00 | 8.08 | Up |
| XP_603981 | DIAPH3 | PREDICTED: diaphanous homolog 3 (Drosophila) | 4 | 2 | 12.26 | 5.85 | Down |
| XP_614269 | FLNA | PREDICTED: filamin A, alpha (actin binding protein 280) | 42 | 57 | 127.16 | 180.91 | Up |
| XP_582024 | HSPG2 | PREDICTED: heparan sulfate proteoglycan 2 | 4 | 0 | 9.37 | 0.00 | Down |
| XP_001253663 | HNRNPM | PREDICTED: heterogeneous nuclear ribonucleoprotein M isoform 2 | 32 | 20 | 126.84 | 81.37 | Down |
| XP_616376 | ITGB3 | PREDICTED: integrin, beta 3 (platelet glycoprotein IIIa, antigen CD61) isoform 1 | 20 | 32 | 61.70 | 108.88 | Up |
| XP_592855 | ILF3 | PREDICTED: interleukin enhancer binding factor 3, 90kDa isoform 2 | 9 | 2 | 32.58 | 8.51 | Down |
| XP_612328 | IARS2 | PREDICTED: isoleucyl-tRNA synthetase 2, mitochondrial | 8 | 1 | 23.25 | 2.27 | Down |
| XP_592952 | MRPL10 | PREDICTED: mitochondrial ribosomal protein L10 | 3 | 0 | 15.51 | 0.00 | Down |
| XP_590721 | NUMA1 | PREDICTED: nuclear mitotic apparatus protein 1 | 7 | 1 | 28.09 | 2.64 | Down |
| XP_614626 | NCL | PREDICTED: nucleolin isoform 1 | 23 | 10 | 69.56 | 27.83 | Down |
| XP_583200 | PCK2 | PREDICTED: phosphoenolpyruvate carboxykinase 2 (mitochondrial) isoform 1 | 27 | 13 | 76.68 | 45.30 | Down |

| | | | | | | | |
|---|---|---|---|---|---|---|---|
| XP_583514 | PGM2 | PREDICTED: phosphoglucomutase 2 | 10 | 4 | 40.83 | 10.55 | Down |
| XP_581432 | SERPINB2 | PREDICTED: plasminogen activator inhibitor-2 isoform 1 | 0 | 10 | 0.00 | 30.23 | Up |
| XP_869492 | SEC23IP | PREDICTED: SEC23 interacting protein isoform 3 | 3 | 3 | 9.91 | 8.86 | Down |
| XP_001788387 | A2ML1 | PREDICTED: similar to alpha-2-macroglobulin-like 1 | 0 | 3 | 0.00 | 8.67 | Up |
| XP_605200 | ATM | PREDICTED: similar to ataxia telangiectasia mutated protein | 4 | 0 | 8.64 | 0.00 | Down |
| XP_001788144 | EDC4 | PREDICTED: similar to autoantigen RCD8 | 3 | 0 | 8.65 | 0.00 | Down |
| XP_001789570 | CPT1A | PREDICTED: similar to carnitine palmitoyltransferase 1C | 7 | 2 | 23.47 | 5.87 | Down |
| XP_870112 | LOC613845 | PREDICTED: similar to chromosome 14 open reading frame 4 | 3 | 0 | 17.39 | 0.00 | Down |
| XP_592923 | CPOX | PREDICTED: similar to coproporphyrinogen oxidase | 8 | 0 | 21.95 | 0.00 | Down |
| XP_001255416 | FMNL1 | PREDICTED: similar to formin-like 1 | 8 | 2 | 21.54 | 4.95 | Down |
| XP_001250990 | HNRNPA3 | PREDICTED: similar to heterogeneous nuclear ribonucleoprotein A3 | 23 | 14 | 71.08 | 41.70 | Down |
| XP_881850 | HNRNPH1 | PREDICTED: similar to Heterogeneous nuclear ribonucleoprotein H (hnRNP H) isoform 26 | 6 | 1 | 21.92 | 4.92 | Down |
| XP_580661 | HNRNPL | PREDICTED: similar to heterogeneous nuclear ribonucleoprotein L isoform 1 | 21 | 12 | 86.03 | 47.24 | Down |
| XP_871851 | HTT | PREDICTED: similar to huntingtin | 0 | 3 | 0.00 | 7.25 | Up |
| XP_585605 | HYOU1 | PREDICTED: similar to hypoxia up-regulated 1 | 55 | 32 | 199.05 | 109.90 | Down |
| XP_001788137 | IRF2BP2 | PREDICTED: similar to interferon regulatory factor 2 binding protein 2, partial | 4 | 0 | 15.65 | 0.00 | Down |
| XP_869445 | MCM2 | PREDICTED: similar to KIAA0030 isoform 3, partial | 12 | 4 | 44.42 | 14.43 | Down |
| XP_001787859 | LRPPRC | PREDICTED: similar to Leucine-rich PPR motif-containing protein, mitochondrial precursor (130 kDa leucine-rich protein) (LRP 130) (GP130) | 12 | 3 | 38.93 | 10.72 | Down |
| XP_001253929 | LOC786695 | PREDICTED: similar to major histocompatibility complex, class II, DR beta 3 | 0 | 3 | 0.00 | 10.34 | Up |
| XP_874112 | MYCN | PREDICTED: similar to N-myc protein | 4 | 0 | 7.57 | 0.00 | Down |
| XP_585195 | OTUD5 | PREDICTED: similar to OTU domain containing 5 isoform 2 | 0 | 6 | 0.00 | 20.06 | Up |
| XP_589061 | PES1 | PREDICTED: similar to Pescadillo homolog 1 | 0 | 4 | 0.00 | 9.89 | Up |
| XP_583607 | PHF12 | PREDICTED: similar to PHD finger protein 12 (PHD factor 1) (Pf1) isoform 1 | 4 | 0 | 8.86 | 0.00 | Down |
| XP_869960 | AIFM1 | PREDICTED: similar to programmed cell death 8 isoform 2 | 6 | 0 | 22.74 | 0.00 | Down |
| XP_606510 | RAB32 | PREDICTED: similar to Ras-related protein Rab-32 | 7 | 2 | 23.43 | 5.37 | Down |
| XP_618140 | EPHA3 | PREDICTED: similar to receptor protein kinase isoform 2 | 0 | 4 | 0.00 | 10.46 | Up |
| XP_582373 | RDH11 | PREDICTED: similar to retinol dehydrogenase 11 isoform 1 | 3 | 0 | 9.47 | 0.00 | Down |
| XP_001254330 | SPCS2 | PREDICTED: similar to signal peptidase complex subunit 2 homolog | 13 | 3 | 43.15 | 9.27 | Down |
| XP_870122 | SF3B1 | PREDICTED: similar to splicing factor 3b, subunit 1 isoform 2 | 6 | 1 | 19.75 | 2.53 | Down |
| XP_618236 | LOC538044 | PREDICTED: similar to T08G11.3 | 24 | 11 | 27.17 | 11.89 | Down |

| | | | | | | | |
|---|---|---|---|---|---|---|---|
| XP_586422 | THUMPD2 | PREDICTED: similar to THUMP domain containing 2 | 8 | 4 | 19.13 | 8.98 | Down |
| XP_001787498 | TM9SF2 | PREDICTED: similar to Transmembrane 9 superfamily member 2 precursor (p76), partial | 10 | 4 | 35.45 | 14.02 | Down |
| XP_001252942 | TREML1 | PREDICTED: similar to Trem-like transcript 1 protein precursor (TLT-1) (Triggering receptor expressed on myeloid cells-like protein 1) | 8 | 0 | 28.19 | 0.00 | Down |
| XP_871340 | UGGT1 | PREDICTED: similar to UDP-glucose ceramide glucosyltransferase-like 1 isoform 2 | 41 | 23 | 140.35 | 79.62 | Down |
| XP_588094 | SPTBN1 | PREDICTED: spectrin, beta, non-erythrocytic 1 isoform 1 | 23 | 7 | 96.24 | 36.08 | Down |
| XP_001250862 | TLN1 | PREDICTED: talin 1 | 15 | 6 | 54.20 | 17.59 | Down |
| XP_614963 | LYN | PREDICTED: v-yes-1 Yamaguchi sarcoma viral related oncogene homolog | 4 | 14 | 13.70 | 40.97 | Up |
| NP_001069141 | PCYOX1L | prenylcysteine oxidase-like precursor | 3 | 0 | 12.97 | 0.00 | Down |
| NP_001029744 | PHB | prohibitin | 13 | 5 | 37.97 | 13.33 | Down |
| NP_001039663 | PHB2 | prohibitin-2 | 11 | 6 | 37.21 | 17.58 | Down |
| NP_001076978 | PCCA | propionyl-Coenzyme A carboxylase, alpha polypeptide | 9 | 3 | 30.66 | 8.35 | Down |
| NP_001033628 | PSMB1 | proteasome beta 1 subunit precursor | 3 | 0 | 8.31 | 0.00 | Down |
| NP_001029516 | ETHE1 | protein ETHE1, mitochondrial precursor | 49 | 32 | 191.18 | 122.56 | Down |
| NP_001099108 | PRPF8 | PRP8 pre-mRNA processing factor 8 homolog | 0 | 3 | 0.00 | 7.22 | Up |
| NP_001069119 | PDXK | pyridoxal kinase | 0 | 5 | 0.00 | 14.93 | Up |
| NP_001030512 | PDHB | pyruvate dehydrogenase (lipoamide) beta precursor | 6 | 1 | 21.07 | 3.95 | Down |
| NP_001094516 | PDHA1 | pyruvate dehydrogenase E1 alpha 1 precursor | 5 | 1 | 21.62 | 3.53 | Down |
| NP_001032700 | G3BP1 | ras GTPase-activating protein-binding protein 1 | 3 | 0 | 8.39 | 0.00 | Down |
| NP_001096578 | RAVER1 | RAVER1 | 6 | 2 | 24.01 | 4.19 | Down |
| NP_001095381 | RCC2 | regulator of chromosome condensation 2 | 15 | 7 | 47.97 | 19.69 | Down |
| NP_872598 | RTN3 | reticulon-3 | 16 | 5 | 44.43 | 17.33 | Down |
| NP_777019 | RNASE6 | ribonuclease K6 precursor | 4 | 0 | 11.31 | 0.00 | Down |
| NP_001076074 | RPN1 | ribophorin I | 57 | 36 | 219.40 | 140.18 | Down |
| NP_001029535 | RBM3 | RNA binding motif protein 3 | 1 | 6 | 4.69 | 23.95 | Up |
| NP_001068643 | SEC11C | SEC11 homolog C | 3 | 0 | 9.19 | 0.00 | Down |
| NP_001095640 | SCAMP2 | secretory carrier membrane protein 2 | 5 | 0 | 21.16 | 0.00 | Down |
| NP_001029454 | SHMT2 | serine hydroxymethyltransferase 2 (mitochondrial) precursor | 50 | 35 | 185.65 | 130.89 | Down |
| NP_803450 | TF | serotransferrin precursor | 0 | 5 | 0.00 | 17.54 | Up |
| NP_001077177 | SRRT | serrate RNA effector molecule homolog | 4 | 0 | 10.68 | 0.00 | Down |
| NP_851335 | ALB | serum albumin precursor | 142 | 90 | 433.87 | 278.56 | Down |
| NP_776882 | SARS2 | seryl-tRNA synthetase 2 precursor | 13 | 7 | 51.98 | 26.39 | Down |
| NP_001096572 | SPN | sialophorin | 7 | 2 | 25.81 | 7.51 | Down |

| | | | | | | | |
|---|---|---|---|---|---|---|---|
| NP_777028 | SLC2A3 | solute carrier family 2, facilitated glucose transporter member 3 | 1 | 6 | 2.35 | 20.80 | Up |
| NP_001094664 | SLC25A12 | solute carrier family 25, member 12 | 11 | 2 | 48.40 | 9.70 | Down |
| NP_001075083 | SF1 | splicing factor 1 | 8 | 3 | 33.84 | 13.82 | Down |
| NP_001096741 | SF3B2 | splicing factor 3b, subunit 2, 145kDa | 4 | 0 | 11.19 | 0.00 | Down |
| NP_001029490 | SFRS2 | splicing factor, arginine/serine-rich 2 | 2 | 4 | 3.59 | 16.85 | Up |
| NP_001032562 | SLIRP | SRA stem-loop-interacting RNA-binding protein, mitochondrial precursor | 8 | 1 | 23.98 | 1.75 | Down |
| NP_991353 | SND1 | staphylococcal nuclease domain-containing protein 1 | 12 | 6 | 46.42 | 15.95 | Down |
| NP_001030559 | NSDHL | sterol-4-alpha-carboxylate 3-dehydrogenase, decarboxylating | 4 | 1 | 12.10 | 1.82 | Down |
| NP_001029696 | HSPA9 | stress-70 protein, mitochondrial precursor | 44 | 28 | 144.73 | 93.21 | Down |
| NP_001030486 | STIM1 | stromal interaction molecule 1 precursor | 7 | 1 | 21.50 | 3.89 | Down |
| NP_001094511 | SSRP1 | structure specific recognition protein 1 | 6 | 1 | 16.81 | 2.26 | Down |
| NP_001091039 | STT3B | STT3, subunit of the oligosaccharyltransferase complex, homolog B | 3 | 0 | 11.89 | 0.00 | Down |
| NP_776603 | SDHA | succinate dehydrogenase ubiquinone] flavoprotein subunit, mitochondrial precursor | 61 | 48 | 217.21 | 160.70 | Down |
| NP_001030254 | SUCLG1 | succinate-CoA ligase, GDP-forming alpha subunit precursor | 43 | 22 | 126.10 | 65.92 | Down |
| NP_001029811 | SUCLG2 | succinate-CoA ligase, GDP-forming beta subunit precursor | 28 | 15 | 100.52 | 67.12 | Down |
| NP_001035601 | SQRDL | sulfide dehydrogenase like | 48 | 30 | 163.55 | 103.02 | Down |
| NP_963285 | SOD2 | superoxide dismutase 2, mitochondrial precursor | 50 | 307 | 201.86 | 1229.62 | Up |
| NP_001039350 | TAPBP | tapasin | 46 | 22 | 156.65 | 72.15 | Down |
| NP_777050 | TXNRD1 | thioredoxin reductase 1 | 1 | 8 | 3.03 | 29.39 | Up |
| NP_001092640 | TMX4 | thioredoxin-related transmembrane protein 4 | 14 | 5 | 44.10 | 14.65 | Down |
| NP_776621 | THBS1 | thrombospondin 1 precursor | 5 | 11 | 18.66 | 44.58 | Up |
| NP_001091474 | TMPO | thymopoietin-2 | 18 | 9 | 61.46 | 29.19 | Down |
| NP_001028933 | TLR7 | toll-like receptor 7 | 5 | 0 | 13.90 | 0.00 | Down |
| NP_001092528 | TOR1A | torsin A | 5 | 1 | 19.52 | 3.48 | Down |
| NP_001095318 | TGM3 | transglutaminase 3 | 0 | 20 | 0.00 | 68.87 | Up |
| NP_001029938 | TMCO1 | transmembrane and coiled-coil domain-containing protein 1 | 3 | 0 | 11.27 | 0.00 | Down |
| NP_001153290 | TMED2 | transmembrane emp24 domain trafficking protein 2 | 7 | 2 | 22.83 | 7.32 | Down |
| NP_001039822 | TMEM173 | transmembrane protein 173 | 10 | 3 | 31.26 | 9.55 | Down |
| NP_001091527 | TAP1 | transporter 1, ATP-binding cassette, sub-family B | 18 | 6 | 70.72 | 23.27 | Down |
| NP_776848 | PLAUR | urokinase plasminogen activator surface receptor precursor | 0 | 7 | 0.00 | 22.43 | Up |
| NP_001030222 | VTN | vitronectin | 0 | 16 | 0.00 | 57.64 | Up |
| NP_776911 | VDAC2 | voltage-dependent anion-selective channel protein 2 | 19 | 13 | 65.04 | 43.74 | Down |
| NP_001039811 | WDR1 | WD repeat-containing protein 1 | 7 | 18 | 28.15 | 70.96 | Up |
